# HomoMINT: an inferred human network based on orthology mapping of protein interactions discovered in model organisms

**DOI:** 10.1186/1471-2105-6-S4-S21

**Published:** 2005-12-01

**Authors:** Maria Persico, Arnaud Ceol, Caius Gavrila, Robert Hoffmann, Arnaldo Florio, Gianni Cesareni

**Affiliations:** 1 Department of Biology, University of Rome Tor Vergata, Via della Ricerca Scientifica 00133 Rome, Italy; 2 Computational Biology Center, Memorial Sloan-Kettering Cancer Center, 1275 York Avenue. New York, NY, USA

## Abstract

**Background:**

The application of high throughput approaches to the identification of protein interactions has offered for the first time a glimpse of the global interactome of some model organisms. Until now, however, such genome-wide approaches have not been applied to the human proteome.

**Results:**

In order to fill this gap we have assembled an inferred human protein interaction network where interactions discovered in model organisms are mapped onto the corresponding human orthologs. In addition to a stringent assignment to orthology classes based on the InParanoid algorithm, we have implemented a string matching algorithm to filter out orthology assignments of proteins whose global domain organization is not conserved. Finally, we have assessed the accuracy of our own, and related, inferred networks by benchmarking them against i) an assembled experimental interactome, ii) a network derived by mining of the scientific literature and iii) by measuring the enrichment of interacting protein pairs sharing common Gene Ontology annotation.

**Conclusion:**

The resulting networks are named HomoMINT and HomoMINT_filtered, the latter being based on the orthology table filtered by the domain architecture matching algorithm. They contains 9749 and 5203 interactions respectively and can be analyzed and viewed in the context of the experimentally verified interactions between human proteins stored in the MINT database. HomoMINT is constantly updated to take into account the growing information in the MINT database.

## Background

The dynamic assembly of stable or transient protein complexes regulates cell physiology by presiding over basic cell functions. In principle, if we knew the kinetic details of the interaction between any macromolecule in a cell, as well as the concentration of each player, we could start thinking about modeling a virtual cell in order to understand, or infer, its response to any given stimulus.

Regrettably we are very far from this level of understanding of the interactions within a cell proteome. In recent years, however, high throughput approaches based on the yeast two hybrid [1] and TAP TAG [2] methods have provided for the first time a genome-wide perspective of the interactome of simple model organisms such as *H. pylori*[3], *E. coli*[4], *S. cerevisiae *[5-8], *C. elegans *[9] and *D. melanogaster*[10,11]. Comparative analysis of comprehensive experiments conducted by different groups, using similar or orthogonal approaches, has led to the recognition that the available interactomes are noisy and largely incomplete [12]. Nevertheless this remarkable experimental effort has put us in a position to analyze the interactomes' broad structure and to start mapping, in these complex protein meshes, the pathway representation we are used to. Unfortunately no such high-throughput data are yet available for the human proteome while genome-wide approaches aimed at the elucidation of the human interactome are only at their inception. However, assuming that functional protein interactions are conserved in evolution, one can consider extending the experimentally determined human protein interaction network by using data from the model organism protein interaction datasets. This can be achieved by transferring the interaction information from each organism to the human proteome and requires the identification of genes that have a common ancestor and share the same function in the two organisms (orthologs). Lehner and Fraser [13] have used the InParanoid algorithm [14] to infer a network of over 70000 interactions between 6200 human proteins generated by using data from the yeast, fly and worm interactome. More recently Brown and Jurisica [15] have developed OPHID a web-based database containing 23359 predicted interactions between human proteins. OPHID was assembled by mapping model organism PPIs to human orthologs using BLASTP and the reciprocal best hit approach. Here we present HomoMINT containing 9749 inferred interactions between 4125 human proteins. We also used the InParanoid algorithm to assign proteins to orthology groups. Whenever two proteins shown to interact in model organisms could be confidently assigned to orthology groups containing a human ortholog, the corresponding main human orthologs (not paralogs) are included in the inferred HomoMINT network. HomoMINT is essentially an 'orthology table' in the MINT database[16]. Thus the inferred network can be freely and conveniently analyzed in the context of the MINT protein interaction data with the aid of the MINT search and analysis tools. HomoMINT is updated daily to take into account the growing number of interactions that are curated each day in the MINT database.

.

## Results

### HomoMINT

Our strategy starts by assigning proteins to orthology groups having a human protein as the main ortholog. An interaction between human proteins is then inferred if both partners of an interaction experimentally verified in model organisms have at least one human ortholog.

Similarly to Lehner and Fraser [13], we have used the InParanoid algorithm to assemble orthology groups. This algorithm has the potential to distinguish between out-paralog, homologous genes that arose by duplication before the speciation event (unlikely to share function), and in-paralogs arising after speciation. However, to avoid unnecessary graphical overcrowding, in the resulting inferred human network (HomoMINT) we have only included interactions between the main human orthologs of each orthology group. An extended network in which the model organism interactions are mapped to all the possible combinations of in-paralogs is also available (HomoMINT_extended). Since InParanoid attributes a score to each orthology assignment it is relatively easy to obtain different inferred networks using orthology tables with varying levels of stringency for assignment to orthology classes.

In addition we have tuned the orthology assignments by imposing the condition that proteins in the same orthology group must have the same domain architecture. This filtering step evaluates the overall protein similarity and eliminates any incongruity caused by the local nature of the BLAST algorithm. Motivated by the observation that multidomain proteins, sharing an exact domain architecture, have significantly higher functional conservation [17,18], we developed a workflow (see Methods) to produce a "high confidence" orthology table in which all orthology group members share the same domain architecture. This filtering procedure improves the functional coherence within the orthology groups (see Methods) while removing only 10% of the 16531 inferred groups. We call the resulting network HomoMINT_filtered.

### HomoMINT as a web server

**Figure 1:**
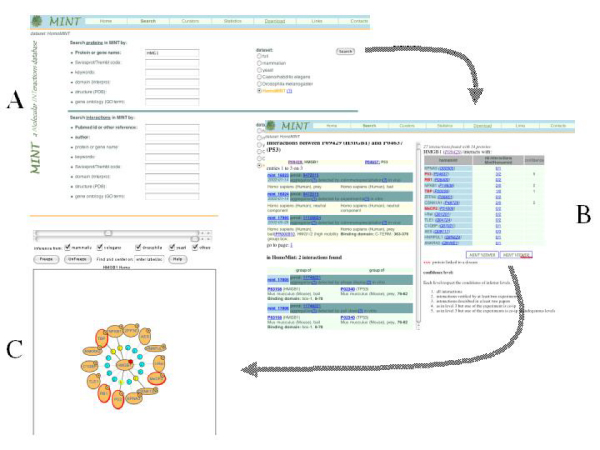
**HomoMINT as a web tool**. HomoMINT can be searched and analyzed by taking advantage of the tools developed for MINT. A) A search can be carried out in the protein table by entering in the form one of the following: a protein name, a Uniprot or a PDB identifier, a keyword, an InterPro domain or a gene ontology term (top part of the form). Alternatively the search can be carried out on the interaction table (centre). Finally (lower part) a BLAST search can be carried out by entering a protein sequence. B) Search output listing on the right the partners of the query protein and on the left the experimental evidence supporting the interactions. C) The Mint Viewer is an applet that permits the graphic display of interaction networks. Edges marked by small blue circles indicate that the corresponding interactions were inferred from experiments carried out in model organisms, while yellow circles mark interactions supported by direct experimental results. Interactions that are inferred from model organisms but are also supported by direct experiments are marked by yellow circles with a blue contour. A series of check boxes make it possible to visualize interactions inferred by any combination of model organism interactomes.

The inferred HomoMINT network has been incorporated into the MINT database [16]. In essence HomoMINT is a calculated table integrated in the MINT relational database. The table is calculated every day by using the orthology group table to map onto the human proteome the interactions that are curated daily in the MINT database. As a result HomoMINT is a dynamic dataset continuously updated that can make use of the search and analysis tools developed for MINT. By entering a protein name, in the MINT search form, one can either perform the search over the experimentally verified interactions between human proteins, as curated in the MINT database, or extend the search to the HomoMINT table, by checking the appropriate radio button (Fig. 1A).

In the latter case one obtains, as a result of the query, both the experimentally verified interactions and the inferred ones. Appropriate links make it possible to retrieve information about the experiments supporting the interaction either directly (experiments carried out with human proteins) or indirectly (experiments carried out in model organisms) (Fig. 1B).

During any MINT search session it is possible to extend the analysis to HomoMINT, by clicking the HomoMINT hyperlink. The composition of the orthology groups used to infer the human interactions can also be inspected via the 'orthology table' hyperlink. A distinction is made between main orthologs (orthologs) and co-orthologs (in-paralogs).

Finally the HomoMINT network can be analyzed, expanded, edited in the context of the experimentally verified protein interactions in the MINT database by using the MINT viewer tool (Fig. 1c). For instance the MINT viewer makes it possible, by checking appropriate boxes, to visualize only interactions inferred from any combination of model organism interactomes. The network visualized and edited by the viewer tool can be downloaded in any of three formats: flat file, XML PSI [19], or in a format that can be used as input for the OSPREY visualization software [20].

### Intersection of HomoMINT with the Human experimental network

Several low throughput experiments, providing evidence of protein interactions between human proteins, have been published in the scientific literature over the past decades. This dataset is approximately the same size as the datasets obtained from the results of high throughput experiments carried out in model organisms, although it is not readily accessible. Recently, a number of databases have started to capture this information and release it in a computer readable format according to a common standard [19]. By merging all the interactions currently deposited in seven major databases [16,21-25], we have assembled a human interactome of 28531 non-redundant interactions. In Table 1 we have reported the analysis of the overlap between the data curated by the different databases.

This assembled human experimental network (HEN) is likely to have some bias in the coverage of the interaction space due to the interest of the scientific community in investigating specific biological domains or to a biased selection of the journal articles curated by the databases. Nevertheless it represents the most accurate representation of the human interactome to date. We used HEN as a benchmark for the initial assessment of the accuracy and the information content of HomoMINT and related inferred networks (Table 2). The networks inferred by Brown and colleagues [15] and by Lehner and Fraser [13] are here referred to as "OPHID" and "Sanger" respectively. As proposed by Marcotte and colleagues [26] we used a unified scoring scheme to evaluate the ability of each inferred network to reconstruct the reference network. To evaluate a dataset we calculated a log likelihood ratio as



where P(I|D) and P(~I|D) are the frequencies of interactions, in a given dataset (D), that are or are not observed in the benchmark dataset (I), while P(I) and P(~I) represent the prior expectations (the frequency of all benchmark gene pairs that do or do not interact).

The overlap between the human experimental network and the one inferred from model organisms (HomoMINT) is 694 interactions (Table 3). This corresponds to 7.1% of HomoMINT, suggesting that both networks only cover a small fraction of the real interactome and that either or both are affected by a large number of false positives. Most of the HomoMINT network (94%) is inferred from interactions that have been obtained by high throughput experiments while only 6% is inferred from higher confidence experiments. Interestingly, the set of high confidence interactions covers more than 26% of the intersection between HomoMINT and the experimental network. The OPHID and Sanger networks are larger since their inference is based on a larger dataset, including computationally predicted interactions datasets (Sanger), and binary interactions, within complexes, being represented by the matrix [27] model (OPHID). This results in a much larger number of binary interactions than for instance those present in networks based on the 'spoke' model. As a consequence the coverage of the HEN network is also larger but the percentage of confirmed interactions and the LLR is lower when compared with HomoMINT. The Sanger core dataset, whose inference is based on a subset of high confidence interactions, is more accurate as is the HomoMINT high confidence network containing only interactions that are inferred when supported by at least two experiments. The highest log likelihood ratio is achieved by a rather limited network HMINT_2org (126 edges) where we have only considered the interactions confirmed by experiments in at least two model organisms. The overlap between the human experimental network and HomoMINT_filtered, obtained by considering only ortholog pairs sharing the same domain architecture, is 453 interactions; these corresponding to almost 9% of the inferred interactions.

### Intersection of HomoMINT with the iHOP resource

The PubMed resource, containing more than 15 million biomedical abstracts, is a valuable resource for high quality protein interactions. As a whole, concurring proteins in PubMed sentences can be considered and modeled as a literature network, which can be superimposed on experimental interaction data or on putative relationships, making it possible to compare new and existing knowledge possible. Here we have made use of a novel text-mining resource, called iHOP (Information Hyperlinked over Proteins) [28] as an independent assessment of the protein interactions predicted in HomoMINT. The iHOP system currently contains 6 million sentences from PubMed abstracts and about 40000 different proteins from human, mouse, and other common animal models (iHOP, ).

Table 4 summarizes the results obtained from this comparison. In particular, we were able to identify a corresponding sentence in the iHOP network for 6.8 % of our predicted interactions. Moreover, 3 % of these sentences expressed the interaction in an explicit protein-verb-protein syntax. In the control set (H_MINT ctrl)), derived from a process of scrambling of the true dataset, less than 1 % of the putative interactor pairs were supported by co-occurrence in sentences in the iHOP database. For comparison the overlap of the iHOP human protein interaction network with our assembled experimental PPI dataset was estimated to be about 22%. Only sentences of high precision were used for the assessment; sentences were excluded from the comparison, when ambiguities between protein-synonyms from different organisms (e.g. Mtx2 in mouse and MTX2 in human) could not be resolved.

For this comparisons we mapped all the proteins to Locus Link ids. In this process proteins (and their interactions) that could not be confidently mapped were eliminated from the networks. For this reason, H_MINT in Table 4 contains 7658 interactions.

### Interacting proteins sharing GO terms

**Figure 2:**
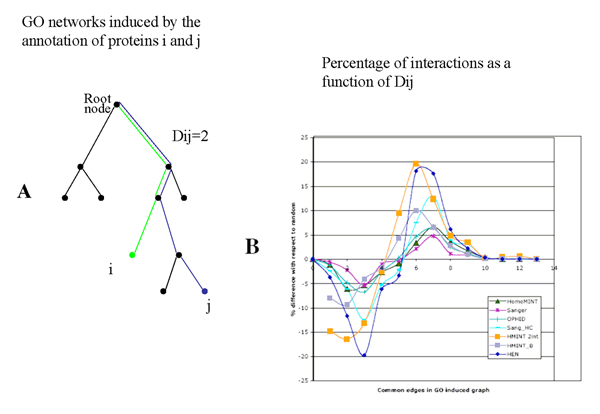
**Degree of common annotation in interacting protein pairs in experimental and inferred networks**. A) Schematic representation of the algorithm used to evaluate the relatedness of gene ontology annotation. The Gene Ontology graph induced by protein 'i ' is in green, while the one induced by protein 'j' is in blue. Dij is the number of edges that the two induced graphs have in common. B) For any given network we have derived a 'scrambled network' containing the same protein nodes linked by the same number of edges with their connections rearranged at random. For each interacting protein pair, in which both proteins have a GO annotation, we have then calculated Dij. Finally we have plotted, as a function of Dij, the difference between the percentage of nodes having a specific Dij in the inferred and in the scrambled network.

The extent of shared annotation in a protein interaction dataset has been previously shown to correlate with accuracy [12,26]. Thus, as a third benchmark for the assessment of the different inferred networks, we estimated the similarity of the Gene Ontology annotation (Biological Process) [29] of any pair of interacting proteins. To determine the relatedness of two GO terms we used the simLL function of the GOstats Package of Bioconductor [30]. This algorithm, as schematically illustrated in Figure 2A, compares the GO graphs 'induced' by two proteins (i, j) and counts the number of edges that are in common between the minimal paths linking the two GO annotation nodes and the ontology root nodes. This value, Dij, is taken as a measure of annotation relatedness. Figure 2B reports, as a function of Dij, the difference between the percentage of interaction pairs showing a given level of GO annotation similarity in an inferred network and in a comparable randomized network. In the randomized network the interactions between the same nodes were reassigned at random. All the inferred networks show a significant difference as compared to the scrambled networks, with the function peaking at Dij = 6 or 7. As was observed in the previous assessment tests, the HomoMINT and OPHID networks perform better than the Sanger dataset, while the Sanger high confidence curve is more similar to the curve of the experimental network. A higher peak at Dij = 7 is observed in the curve of HomoMINT_filtered, obtained by filtering the orthology groups to remove proteins displaying a different protein architecture, or in the curve of HMINT_2int, a high confidence network obtained by considering only interactions supported by at least two experiments.

### HomoMINT as a graph

**Figure 3:**
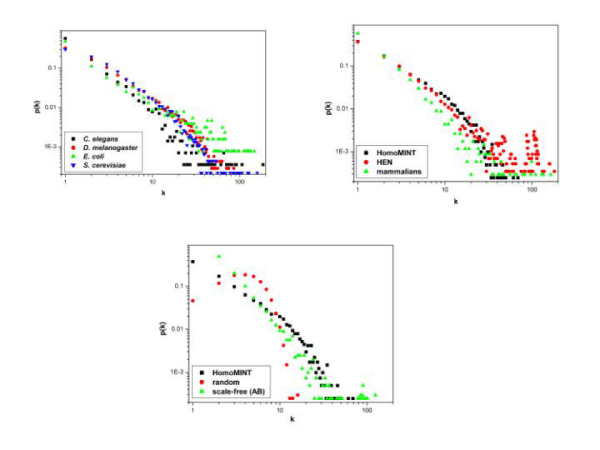
**Degree distribition of the HomoMINT network compared with different biological networks**. Frequency of nodes with k links for A) the model organism experimental networks in the MINT database B) the assembled Human experimental network (HEN), the Human inferred (HomoMINT) data set, the Mammalian data set in MINT and C), for a random network of similar size and for a scale-free network assembled according to Barabasi [31].

Protein interaction networks can be described as graphs where nodes and edges represent proteins and their interactions respectively. Although, at a first sight, apparently random in their topology, biological networks are characterized by a number of properties differentiating them from random networks. Specifically they have a large average clustering coefficient [31]. Most remarkably the distribution of protein connectivity is scale-free. As shown in Figure 3 the HomoMINT network, as well as the assembled human interaction network, has a scale-free topology with its degree distribution not differing substantially from those of the interactomes of model organisms.

In Table 5 we have reported the analysis of some characteristics of the HomoMINT graph and we have compared them with those of some experimental networks in the MINT database. In HomoMINT the average clustering coefficient, the parameter that most captures the modularity of biological networks, is considerably higher than that of a random network of similar size and is consistent with the values found in biological networks. Also the remaining parameters describing the HomoMINT graph are typical of biological networks.

## Discussion

Several databases, using a variety of computational methods to make inferences about functional relationships between genes and proteins, are available on the web [32-35]. HomoMINT is an inferred human protein network obtained by transferring the experimental interaction annotation from the proteome of seven model organisms to the corresponding ortholog human proteins. The orthology mapping is obtained by means of the InParanoid algorithm.

Approximately one fifth of the interactions present in the MINT database could be mapped to human orthologs thus resulting in the assembly of an inferred network linking 4125 human proteins with 9749 edges. While a large proportion of these proteins are not functionally annotated one can use HomoMINT to transfer functional information from better characterized neighbors in the graph.

Because of evolutionarily frequent molecular processes leading to gene family expansion or contraction, the transfer of interaction information between organisms, especially high eukaryotes, is complicated by the abundance of paralogs in orthology groups. The InParanoid algorithm is designed to distinguish paralogs arising before or after speciation events. We have chosen to transfer the interaction information only to the main human ortholog in each group. Thus our inferred network is essentially based on orthology mapping by the reciprocal best hit approach. However, the orthology groups assembled in our web available table contain paralogs, so permitting any alternative choice. Furthermore since the InParanoid algorithm provides a confidence score for each orthology assignment the likelihood of the inferred interactions can be evaluated from the confidence score of the model organism and human gene orthology assignment as proposed for instance by Lehner and Fraser [13].

To assess the predictive value of HomoMINT, we performed a number of tests aimed at assessing to what degree of accuracy and coverage the orthology based inferred networks could be supported by previous knowledge. We first assembled a human experimental network from the protein interaction data stored in PPI databases and determined the percentage overlap between this network and HomoMINT or related networks. Next, we estimated the enrichment in the inferred networks of interacting proteins sharing Gene Ontology annotation. Finally we estimated the overlap between the inferred networks and the iHOP literature network.

Our approach is based on the assumption that protein interactions between ortholog proteins are conserved in evolution. To what extent this is true cannot at present be estimated because of the incompleteness and inaccuracy of the available experimental datasets [36]. Even hypothesizing that the assumption is 100% correct, the accuracy and coverage of the inferred network is still limited by the quality of the original model organism interaction datasets and our ability to identify the true human orthologs of a model organism protein. Not surprisingly our benchmark tests show that accuracy increases if one uses more stringent criteria for orthology assignment (for instance by only allowing orthologs with similar modular architecture) or if one bases the inference on a more reliable interaction dataset (for instance relying on multiple evidence).

In contrast with similar projects [13,15,37], HomoMINT is unique for its direct link to a curated PPI database. HomoMINT is a calculated section in the MINT relational database and its content is updated daily to take into account the newly curated entries in the MINT database. Furthermore the MINT viewer makes it possible to analyze and edit the HomoMINT network in the context of the experimentally verified interactions deposited in the MINT database. HomoMINT can be searched and analyzed at . The HomoMINT dataset is available either as a flat file or a PSI XML file (see Additional file 1 and Additional file 2 for details). Each of them contains all interaction inferred from model organism's protein on main human orthologs.

Click here,  and fill the requested fields to have access to the latest release files.

## Conclusion

Since it is not clear which percentage of PPI are conserved through evolution [36] HomoMINT should be considered as a hypothetical network that can be of use in predicting functions of yet uncharacterized proteins, in making experimentally testable hypotheses about new participants in well studied pathways and in prioritizing interactions to be tested in large scale PPI experiments. As such, the network should provide a rich source of functional hypotheses for researchers interested in the functions of one or many human proteins.

## Methods

### Software

BLASTP searches were carried out using blastall 2.2.9 [38].

InParanoid algorithm version 1.35 was downloaded from: .

Graph analysis and GO functional annotation analysis were performed by using R package version 2.0.1 [39] and the Bioconductor modules *graph, RBGL, GOstats *[30].

### Data Sources

The proteome sets for the BLAST searches and ortholog table assembling were downloaded or built from the following sources: *Arabidopsis thaliana *proteome set (predicted proteins), *Caenorhabditis elegans *(predicted proteins), *Drosophila melanogaster *(predicted proteins), *Escherichia coli K12 *(predicted proteins), *Homo sapiens *(predicted proteins), *Mus musculus *(predicted proteins), *Rattus norvegicus *(predicted proteins), *Saccharomyces cerevisiae *(predicted proteins),  Multiple species proteome set (predicted proteins),  by querying the database for proteins belonging to the following species: Sus scrofa (Pig), Xenopus laevis (African clawed frog), Ovis aries (Sheep), Oryctolagus cuniculus (Rabbit),Gallus gallus (Chicken), Canis familiaris (Dog),Bos taurus (Bovine).

### Assembly of the orthology table

The procedure implemented in the InParanoid algorithm [14] starts with an all-against-all BLASTP comparison between two proteomes of interest. Reciprocal best hit criteria are used to identify orthologous relationships between pairs of proteins. For each putative ortholog, probable recent paralogs or in-paralogs are identified as sequences within the same proteome that are reciprocally more similar to each other than either is to any sequence from the other proteome.

An InParanoid confidence level cut-off of 0.6 was chosen for the assignment of in-paralogs to orthology groups. Due to the redundancy of the starting proteome sets, several groups contained identical copies of the same protein. To limit this problem we decided to eliminate paralogs with InParanoid confidence level above 0.98. InParanoid performs its comparison between each pair of proteomes. To build an orthology table with orthology groups including proteins from all organisms of interest, we used python scripts to merge the InParanoid results keeping a human protein as reference for each orthology group.

### Assembling HEN (Human Experimental Network)

The human experimental interactome has been assembled by importing the data in a Postgresql database from the following resources: Intact (XML PSI files),1300 unique interactions at  DIP (Flat file),833 unique interactions at  BIND (XML PSI 2 file),4073 unique interactions at  MINT, 3679 unique interactions at  HPRD (XML PSI file), 6153 unique interactions at  MIPS (XML PSI file), 322 unique interactions at  Only interactions that could be confidently mapped to Uniprot ids were added to HEN.

### Filtering orthology groups for domain architecture homogeneity

A procedure has been developed to improve and to measure the functional coherence in orthology groups, based on dynamic programming techniques and implemented as a string matching algorithm [40].

We modeled every protein in our orthology groups as an ordered string of domains. To this end, we used the domain annotations available in SMART [41] and PFAM [42]. In particular, the human and the other eight model organism proteomes under analysis have been surveyed for their specific domain architectures. Repetitions of the same domain are treated as a single instance of that domain. Overlapping domains are considered as independent elements of the string representing the domain architecture of the protein.

Then we developed a PERL string matching algorithm to establish distances between the proteins in terms of similarities between their domain architectures. Each protein is represented as a string of concatenated ordered domains. Thus we were able to measure a distance between two proteins by counting the number of domain editing steps (deletions, insertions, substitutions) in order to match the domain architecture of the two proteins. Proteins identical in their domain architecture will have an "edit distance" equal to zero. Distances are normalized by dividing for the total number of domains in the ortholog human protein.

This procedure prevents proteins with markedly different domain architecture (and function) from being clustered mistakenly in a group, although they share similarities only within distinct regions of a multidomain protein. In this way we tried to take in account not only local relationships among sequences to be merged in the orthology groups but global relationships as well.

To assess the filtering procedure we examined the consistency of the annotation of the members within each orthology group, as reported in the ENZYME database [43]. We were able to attribute at least two ENZYME annotations to 9% of groups constituting the filtered orthology table. Fewer than 6% of these groups (77/1355) were declared inconsistent with the ENZYME hierarchic classification scheme. 17 inconsistent groups present in the standard orthology table were not present in the filtered orthology table, underlining the improvement of the functional coherence in the orthology groups after filtering for similarity in domain architectures. The number of inconsistent groups in the standard orthology table was 94 out of 1396 groups which have at least two ENZYME annotations.

### Gene Ontology similarity analysis

The algorithm for measuring the Gene Ontology annotation similarity of a pair of proteins is based on the simLL function of the GOstats package of Bioconductor [30]. For each pair of proteins (Pi, Pj) and for each ontology, the function simLL assigns, in three steps, a unique measure of similarity, called Dij:

(1) Finds all the terms to which Pi and Pj are annotated including the parent terms. These sets of terms in the Gene Ontology tree represent the nodes of the GO graphs induced by Pi and Pj, respectively.

(2) Find the set of terms which the GO graphs induced by Pi and Pj have in common. Denote this set Sij.

(3) Define the depth of each term in Sij to be the length of the shortest path between the term and the root node of the ontology (here length refers to number of connecting edges).

(4) Find the maximum depth of terms in the set Sij. We refer to this value as Dij.

## Authors' contributions

MP carried out programming and analysis on ortholog-paralog detection, Gene Ontology annotation, domain architecture matching algorithm and participated in graph theory based network analysis as a Ph.D.student in the lab of GC; she designed the organization and drafted the manuscript.

AC performed the integration of HomoMINT in the enviroment of MINT database; he also assembled the human experimental network and helped to draft the manuscript.

CG and AF developed the software for modeling and generation of random networks, performed network comparison analysis and helped to draft the manuscript. RH was involved in the benchmarking of HomoMINT against iHOP resource. GC supervised the study and wrote the manuscript. All authors read and approved the final manuscript.

## Supplementary Material

Additional File 1 In the homomint-flat file (dump-homomint.txt), the fields are: • the mint id of the interaction it has been infered from • uniprot id of the first human protein • short label for the first protein • uniprot id of the first protein of inference (model organism) • uniprot id of the second human protein • short label for the second protein • uniprot id of the second protein of inference (model organism) • model organism from which the interaction on Human has been infered Click here for file

Additional File 2 In the XML file (homomint-2005-04-30.xml) : • each ProteinParticipant has for first xref the uniprot reference of the human protein and as a secondary xref the ortholog in the model organism (secondary="inferred from") • the xref at the interaction level contains the mint id for the interaction from which ithas been infered • the attribute list on the interaction level contains an attribute "model organism" that contains the organism from which this interaction has been inferred. Click here for file

## Figures and Tables

**Table 1:** Intersection of human interactomes in public databases

		MINT	DIP	BIND	Intact	React.	HPRD	MIPS
	Nr. of edges							
MINT	3679	x	315	340	1350	101	429	54
DIP	990		x	158	22	67	209	26
BIND	4671			x	356	229	733	50
Intact	2860				x	103	208	16
Reactome	15068					x	269	16
HPRD	6891						x	84
MIPS	777							x

**Table 2:** Inferred ad experimental networks compared in this work

Dataset	Number of interactions	Description or reference
OPHID	23359	[15]
Sanger	37007	[13]
Sanger H.C.	5647	[13]
HomoMINT	9749	This work
HomoMINT_filtered	5203	HomoMINT filtered for domain architecture conservation.
HMINT_2 int	290	inferred from interactions confirmed by at least two experiments.
HMINT_2 org	126	inferred from interactions supported by experiments in at least two model organisms
HM_LT	543	Inferred from interactions discovered by low throughput experiments.
HEN	28531	Compilation of interactions between human proteins
iHOP	278452	[28]

**Table 3:** Overlap between inferred and experimental human networks

		OPHID	Sanger	HEN	% overlap	LLR
		23359	37007	28531		
HomoMINT	9749	3501	2794	694	7.1	4.2
OPHID	23359		7067	1632	7.0	4.1
Sanger	37007			1504	4.1	3.6
Sanger H.C.	5647			841	14.9	5.0
HM_filtered	5203	1818	1391	453	8.7	4.4
HMINT_2int	810	290	227	218	26.9	5.7
HMINT_2org	126	70	75	60	47.6	6.6
HM_LT	543	69	63	131	24.1	5.6

For this comparison we mapped all the proteins to Uniprot ids. In this process proteins (and their interactions) that could not be confidently mapped were eliminated from the networks.

**Table 4:** Overlap of the inferred and experimental human networks with iHOP

		H_MINT	H_MINT ctrl	Sanger	Sanger ctrl.	OPHID	OPHID ctrl.	HEN	HEN ctrl.
	Nr of Edges	7658	7658	26590	26590	12887	12887	23332	23332
iHOP* (sentence)	278,452	522 (6.8)	57 (0.7)	857 (3.2)	233 (0.8)	941 (7.3)	88 (0.7)	5293 (22.6)	615 (2.7)
iHOP (pattern)	47,807	229 (3)	9 (0.1)	254 (1)	53 (0.2)	468 (3.6)	14 (0.1)	2675 (11.5)	176 (0.7)

*The iHOP (sentence) network includes interactions between proteins whose names are found in the same sentence in an abstract. iHOP (pattern) is a subnetwork linking proteins found in a pattern of type gene_name_A/verb/ gene_name_B. The networks that are compared with iHOP are described in the main text. The corresponding 'ctrl' networks are scrambled networks containing the same nodes and the same number of edges. For this comparison we mapped all the proteins to Locus Link ids. In this process proteins (and their interactions) that could not be confidently mapped were eliminated from the networks.

**Table 5:** Graph analysis

Data set	Nodes (N)	Edges* (L)	Clust. coeff. 1	MPL2	<k> 3	d_LCC4
HomoMINT	4067	9132	0.04	4.9	4.73	12
HEN	4933	22124	0.16	4.5	9.4	15
C. elegans	2834	4406	0.02	4.8	3.2	13
D. melanogaster	7005	20282	0.01	4.4	5.8	11
S. cerevisiae	4584	12055	0.07	4.4	5.3	12
Random2000 #	1989	5047	0.002	4.8	5.0	11
Random5000 #	4893	9935	0.001	6.2	4.0	13

*Number of edges may be different from those reported in Table 2 because in this analysis we have neglected interactions leading to homodimerization.

^#^Random2000 and Random5000 are random networks with approximately 2000 and 5000 nodes.

^1^Average of the clustering coefficient of the nodes in the network.

^2^MPL is the average of the minimal path length between two nodes of the graph.

^3^<k> is the average number of connections per nodes in the graph.

^4^d_LCC is the diameter of the largest connected component of the graph
